# A Versatile Optical Clearing Protocol for Deep Tissue Imaging of Fluorescent Proteins in *Arabidopsis thaliana*

**DOI:** 10.1371/journal.pone.0161107

**Published:** 2016-08-12

**Authors:** Thomas J. Musielak, Daniel Slane, Christian Liebig, Martin Bayer

**Affiliations:** 1 Max Planck Institute for Developmental Biology, Department of Cell Biology, Spemannstrasse 35, 72076 Tuebingen, Germany; 2 Max Planck Institute for Developmental Biology, Light Microscopy Facility, Spemannstrasse 35, 72076 Tuebingen, Germany; University of Nottingham, UNITED KINGDOM

## Abstract

Confocal microscopy is widely used to visualize gene expression patterns and developmental processes in plants. However, the imaging of plant tissue can be challenging due to its opacity, which often makes previous immersion in a clearing agent necessary. Many commonly-used chemicals suffer either from their incompatibility with fluorescent proteins or their complex and lengthy application. 2,2'-thiodiethanol (TDE) has recently been described as a clearing agent with an emphasis on high resolution microscopy due to its potential to adjust the refractive index. Here, we evaluate the use of TDE-based clearing for confocal as well as two-photon microscopy in various *Arabidopsis thaliana* tissue types. We demonstrate that tissue fixation is a mandatory prerequisite for the use of TDE, in order to preserve tissue integrity and fluorescent protein activity. TDE concentrations between 50–70% are a good compromise for imaging of technically challenging tissue to achieve good clearing without affecting fluorescent protein activity. TDE-based clearing is simple and rapid to use and allows for a flexible experimental setup while facilitating high quality imaging.

## Introduction

Plant cells are surrounded by a rigid cell wall, which gives them a geometrically well-defined shape. In combination with their high water content, plant cells are in principle very suitable for light microscopy. However, microscopy of thicker sections of plant tissue can be challenging, due to high degree of inhomogeneity with respect to refractive indices (RI) of the various inter- and intracellular tissue components. This causes strong scattering of light preventing e.g. deeper fluorescent imaging. In recent years, especially two-photon microscopy had a major impact on plant microscopy and facilitated deep penetration of tissues in combination with fluorescent markers [1]. Although two-photon-microscopy is less prone to light scattering and auto-fluorescence artefacts since it uses near infrared wavelengths for excitation, it is still limited by the earlier mentioned optical inhomogeneities of the sample [2, 3]. To overcome these, several clearing techniques have been developed to achieve an optically homogeneous sample.

For clearing of mammalian tissue, a wide range of chemical agents is commonly available (reviewed in [4, 5]), among them for example urea-based reagents like Scale or CUBIC [6–8], or sugar-based reagents like SeeDB [9]. Solvent-based techniques often rely on the use of hazardous chemicals and cause quenching of fluorophores due to their dehydrating nature [10, 11]. In recent years also hydrogel-based methods have been established, to avoid tissue shrinkage or collapse caused by clearing substances [12, 13]. In summary, most of these approaches have in common that sample preparation is either time-intensive or technically challenging [reviewed in 4].

In plant research, established and commonly-used clearing methods mainly rely on chloral hydrate [14] because of its good clearing abilities and its simple usage. Chloral hydrate has been intensively used for a wide range of plant tissues and appears in a plethora of protocols and variations, e.g. as Hoyer's clearing agent. It has an RI of 1.43 (plant cell wall: 1.42) and makes the plant material more transparent as it reduces light scattering within the sample. While chloral hydrate can successfully be combined with staining methods like aniline blue or GUS staining [15, 16], it unfortunately inactivates fluorescent proteins (FPs) and can therefore not be used in combination with FP imaging. Recently, optical clearing methodologies have also been introduced for plant tissue that allow FP detection. A class of chemicals, which can potentially be used with living tissues, are perfluorcarbons [17, 18]. Their clearing effect, however, is limited due to an RI of around 1.3 and they have so far only been shown to work efficiently with leaf tissue, but supposedly not in other tissue types [5, 17]. Two additional, very potent clearing reagents ClearSee and PEA-CLARITY [19, 20], are improvements of existing hydrogel- and urea-based protocols [12, 21].

2,2’-thiodiethanol (TDE) was used successfully in several studies in combination with fluorescent proteins in animals [22–24]. TDE was discovered in a screen for chemical substances that optimize the refractive index for high resolution microscopy [25]. The refractive index of TDE can be adjusted in a wide range (up to 1.515), thereby allowing for a perfect match between the embedding medium and the specimen of choice. Recently, Hasegawa et al. (2015) introduced TDE as clearing agent for plant samples and demonstrated the detection of fluorescent proteins in tissue embedded in 20% TDE.

Here, we evaluate different tissue fixation times and TDE concentrations to find an optimal compromise between tissue clearing and preserving tissue integrity and fluorescent protein activity in different Arabidopsis tissue types. We demonstrate benefits and limitations of TDE in tissue clearing and optical homogenization of challenging plant organs in combination with the detection FPs by single photon and two-photon microscopy.

## Results

### Tissue clearing properties of TDE

**Fig 1:**
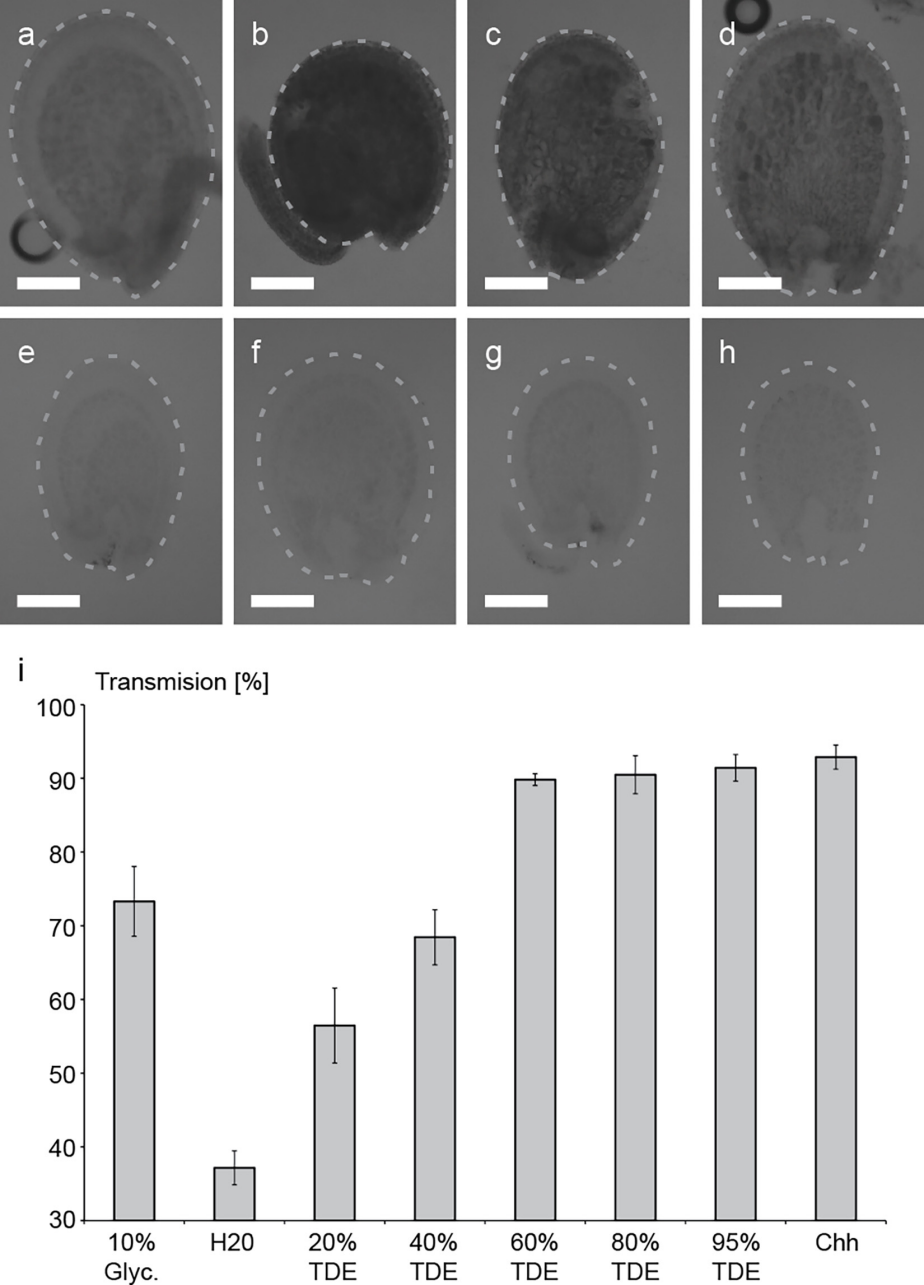
Optical clearing of immature seeds by TDE embedding. (a-h) Brightfield images of whole seeds embedded in different TDE concentrations and control solutions. (a) 10% Glycerol (Glyc.), (b) H2O, (c) 20% TDE, (d) 40% TDE, (e) 60% TDE, (f) 80% TDE, (g) 95% TDE, (h) Chloral hydrate (Chh). Percentage of transmission for the different clearing solutions (i) was calculated by dividing average grey values of seed by average background grey values. Error bars indicate standard deviation of transmission for n = 8 seeds for each clearing solution. Scale bars = 50μm.

Many plant samples are not transparent enough to be readily used for confocal microscopy. Recently, a TDE-based protocol was introduced for clearing of several organs of Arabidopsis (Hasegawa et al. 2016). To evaluate the clearing ability of TDE in plant tissue, we incubated immature Arabidopsis seeds in increasing TDE concentrations and determined afterwards the light transmission through the tissue (Fig 1). Consistent with previous reports by Hasegawa et al., our results indicate that the opacity of the tissue is reduced by TDE in a concentration-dependent manner (Hasegawa et al., 2016). The maximum clearing of ovular tissue was reached between 60–95% TDE in this experiment and no further increase in clearing could be achieved with higher TDE concentrations. The clearing by TDE was not as intensive as with chloral hydrate but was far superior to glycerol (Fig 1). Our results further indicated that for ovular tissue, an incubation time of one hour was already sufficient to achieve a good clearing result, allowing for fast sample preparation.

### TDE can be used in combination with various fluorescent proteins

**Fig 2:**
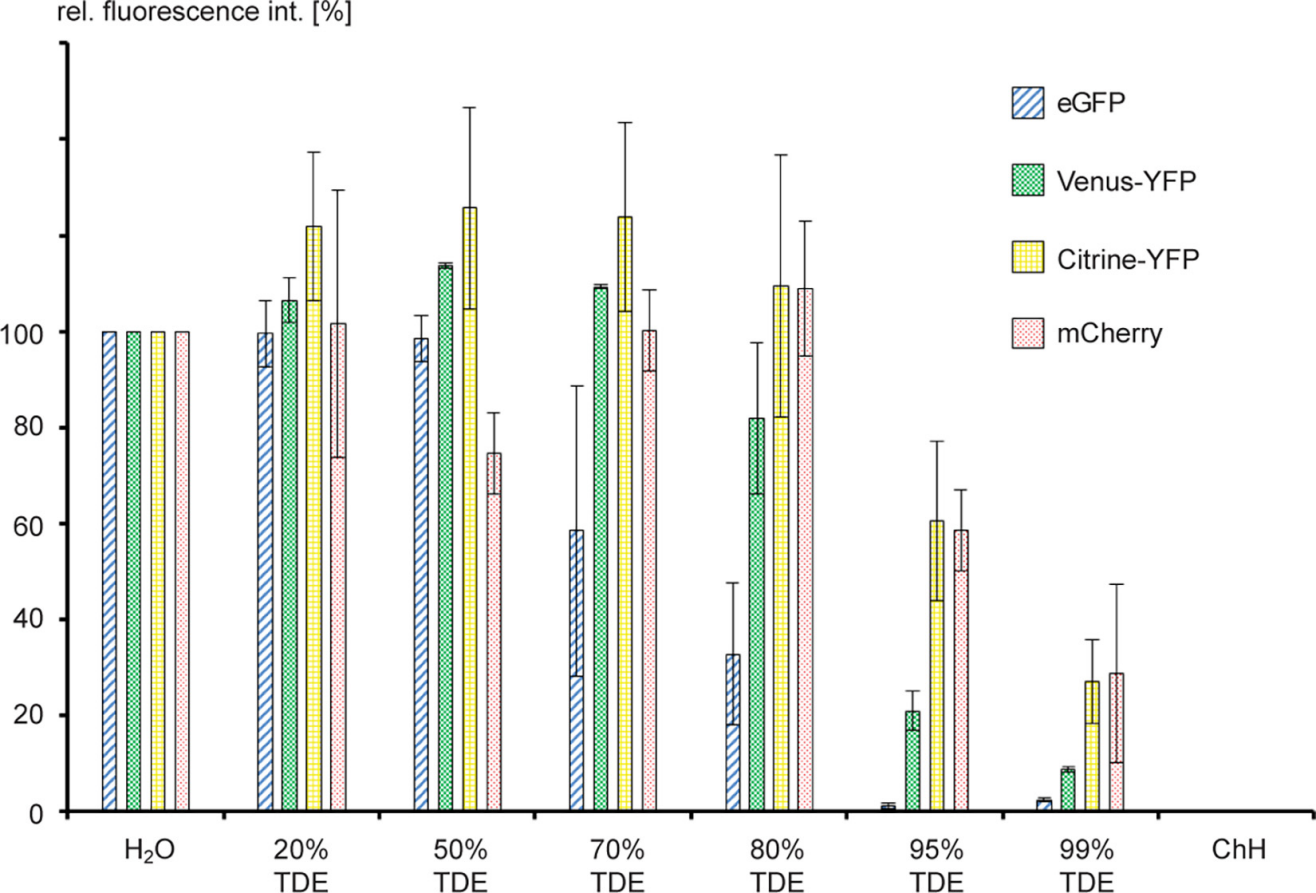
Measurement of fluorescence activity of FPs after incubation of 1h in TDE. Fluorescence of heterologously expressed and purified FPs was measured in vitro. As controls, FPs were incubated in water (H_2_O) as well as in chloral hydrate (ChH). Fluorescence in water was defined as 100%.

It has been shown previously that fluorescent proteins can be detected in TDE-cleared tissue at low (20%) TDE concentrations [24]. For good tissue clearing, however, higher TDE concentrations would be advantageous. As TDE is a hygroscopic substance and common fluorescent proteins rely on the presence of bound water molecules in the fluorophore [26], we wondered if increased TDE concentrations would be deleterious for FP activity. We measured in vitro the fluorescence intensity of various heterologously expressed and purified fluorescent proteins (eGFP, Venus-YFP, mCitrine-YFP, and mCherry-RFP) in increasing concentrations of TDE with different incubation times (Fig 2 and S1 Fig). As controls, we measured the various FPs in water and in chloral hydrate. No fluorescence signal was detectable in the chloral hydrate-containing controls, confirming the notion that chloral hydrate completely inactivates fluorescent proteins. In contrast, incubation in 20–70% TDE lead to higher fluorescence intensities for YFP variants, while there was a decline of fluorescence in TDE concentrations above 95% in comparison to proteins in water for all fluorophores. In 99% TDE, all proteins displayed a strong reduction in fluorescence. eGFP on the other hand, seemed to be the most sensitive fluorophore of the tested fluorescent proteins as there was a decline in fluorescence already noticeable in TDE concentrations greater than 50%. The fluorescent intensities of each fluorescent protein varied very little over the course of 60 min, indicating that the fluorophores were quite stable in TDE (S1 Fig). Taken together, our results show that under in vitro conditions, FPs are functional in TDE concentrations up to 95%, giving the user the possibility to adjust a wide range of refractive indices for the embedding medium. Furthermore, our results suggest that low TDE concentrations can even exert a positive effect on the fluorescence intensity of some FPs.

### TDE can potentially help reducing bleaching effects of tdtomato-RFP

A major problem of confocal microscopy, in particular single-photon microscopy is photo-oxidation or bleaching [27]. An interesting feature of TDE in this context is its reducing ability, suggesting that TDE might be able to suppress bleaching effects [25]. We speculated if the observed increase of fluorescence intensity of some FPs in higher TDE concentrations might be a result of reduced photo bleaching. To test this in a more systematic fashion in situ, we performed a bleaching experiment in roots expressing nuclear-localized eGFP and tdtomato immersed in two different concentrations of TDE, 10% glycerol and water, respectively (S2 Fig). Again, both FPs showed different reactions to the various embedding media. For eGFP, the effect of different embedding media on bleaching was relatively weak. For tdtomato on the other hand, different embedding media lead to noticeable differences in bleaching. Higher TDE concentrations seem to have a positive effect with the best signal retention in 95% TDE and the lowest in water. It has to be kept in mind however, that both fluorescent proteins showed reduced total fluorescence in 95% TDE in comparison to lower TDE concentrations or water before bleaching (Fig 2). Therefore in terms of total fluorescence, the positive effect of reduced bleaching can be masked by the overall reduced fluorescence of the fluorophore. eGFP behaved more similar in different embedding media with respect to bleaching and displayed lowest bleaching in glycerol and water, closely followed by TDE. This finding reflects specific behaviour of each single FP to TDE, an effect that has been reported before [22, 25]. Our results indicate that TDE can in principle help to reduce photo-oxidation of some fluorophores such as tdtomato. However, it is questionable if it makes any significant difference for most imaging experiments since the observed effect is rather weak.

### Tissue fixation is a necessary pre-requisite for TDE clearing

Taking in vitro data and clearing experiments together, our results suggest that concentrations of around 70–80% TDE would be the best compromise to achieve maximal clearing and best conservation of fluorescence signal. However, this was deduced from in vitro data with isolated proteins. We therefore wanted to test if this also holds true for fluorescent proteins expressed in plant tissue.

**Fig 3:**
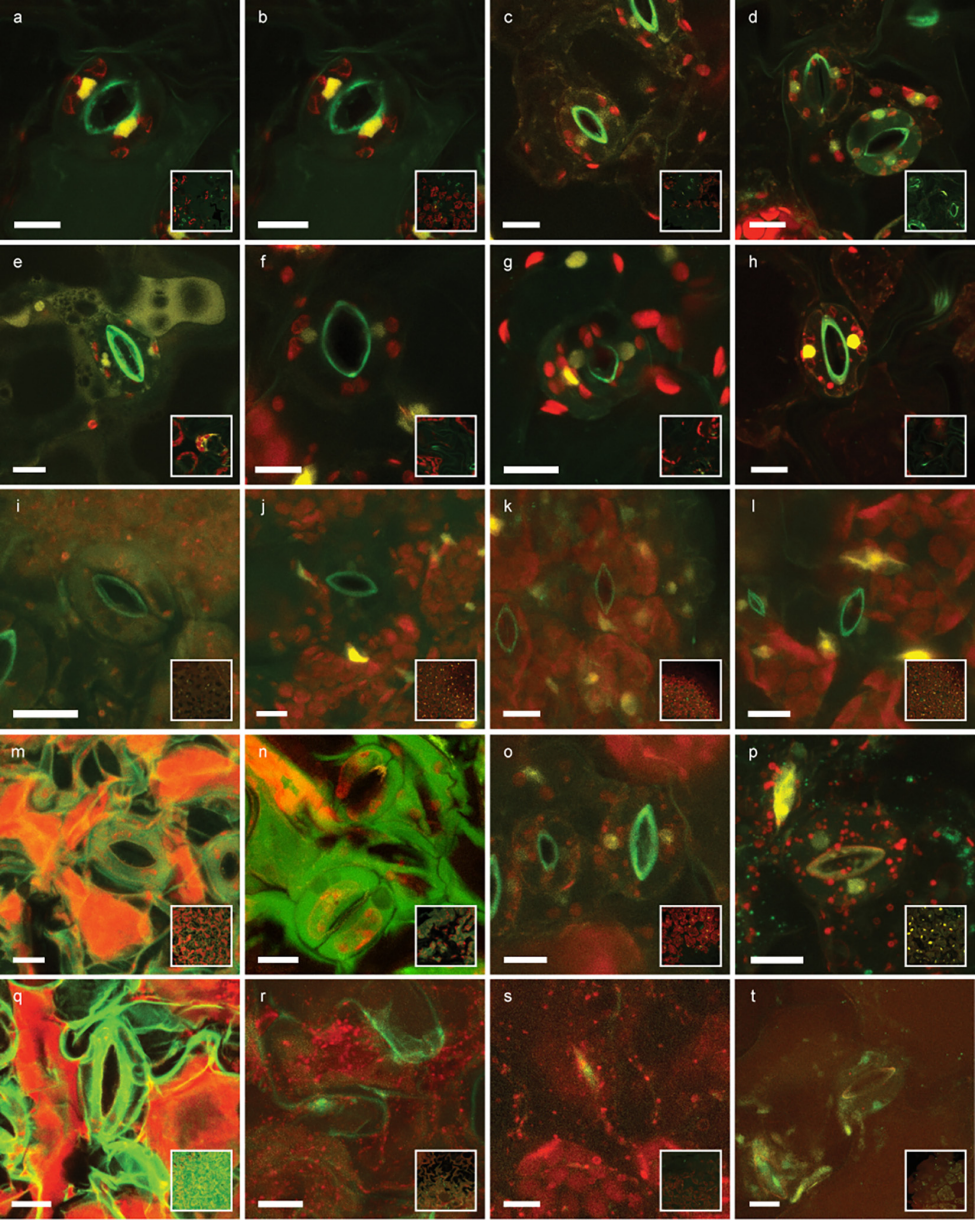
Effect of fixation time, TDE concentrations and incubation time. Cotyledons of 5 day-old seedlings ubiquitously expressing nuclear-localized tdtomato (yellow) were either not fixated (a, e, i, m, q), fixated for one hour (b, f, j, n, r) or fixated overnight (c, d, g, h, k, l, o, p, s, t) in 4% PFA solution. Afterwards samples were incubated for one hour (a-c, e-g, i-k, m-o, q-s) or overnight (d, h, l, p, t) in the respective embedding medium: 10% glycerol (a-d); 20% TDE (e-h); 50% TDE (i-l); 70% TDE (m-p); 95% TDE (q-t). Overview of leaf surface shown as inlay. Autofluorescence excited by 405 nm laser line shown in green, autofluorescence of chlorophyll excited by 552 nm laser line shown in red. Scale bars = 10μm.

We examined ubiquitously expressed nuclear-localized tdtomato (pRPS5a>>NLS-tdtomato) in cotyledons of 5 day old seedlings. To evaluate the effect of fixation time, tissue was either not fixated or fixated for 1h or overnight at 4°C in 4% PFA solution. Afterwards, we incubated the tissue for 1h and overnight in different TDE concentrations or 10% glycerol to test the effect of different incubation times (Fig 3).

Without fixation and with one-hour fixation, lower TDE concentrations can already lead to diffusion of chlorophyll throughout the tissue as well as leakage of NLS-tdtomato out of the nuclei (Fig 3E, 3I and 3J). This would indicate that membrane structures might be compromised by TDE if the tissue is not fixated sufficiently. Furthermore, higher TDE concentrations lead to tissue shrinkage and deformation and loss of tdtomato fluorescence (Fig 3N and 3Q–3T). For 20% TDE, already a short fixation of one hour was sufficient to preserve FP activity and tissue integrity (Fig 3F). For higher TDE concentrations, overnight fixation was necessary to avoid negative effects of TDE incubation. TDE concentrations of 95% lead to strong tissue shrinkage as well as loss of FP activity even after previous overnight fixation of the tissue (Fig 3S and 3T). Therefore, for leaf tissue overnight fixation and incubation in 50% TDE seemed to be the clearing method of choice to obtain a good compromise between tissue clearing and conservation of tissue integrity and fluorescent protein activity.

**Fig 4:**
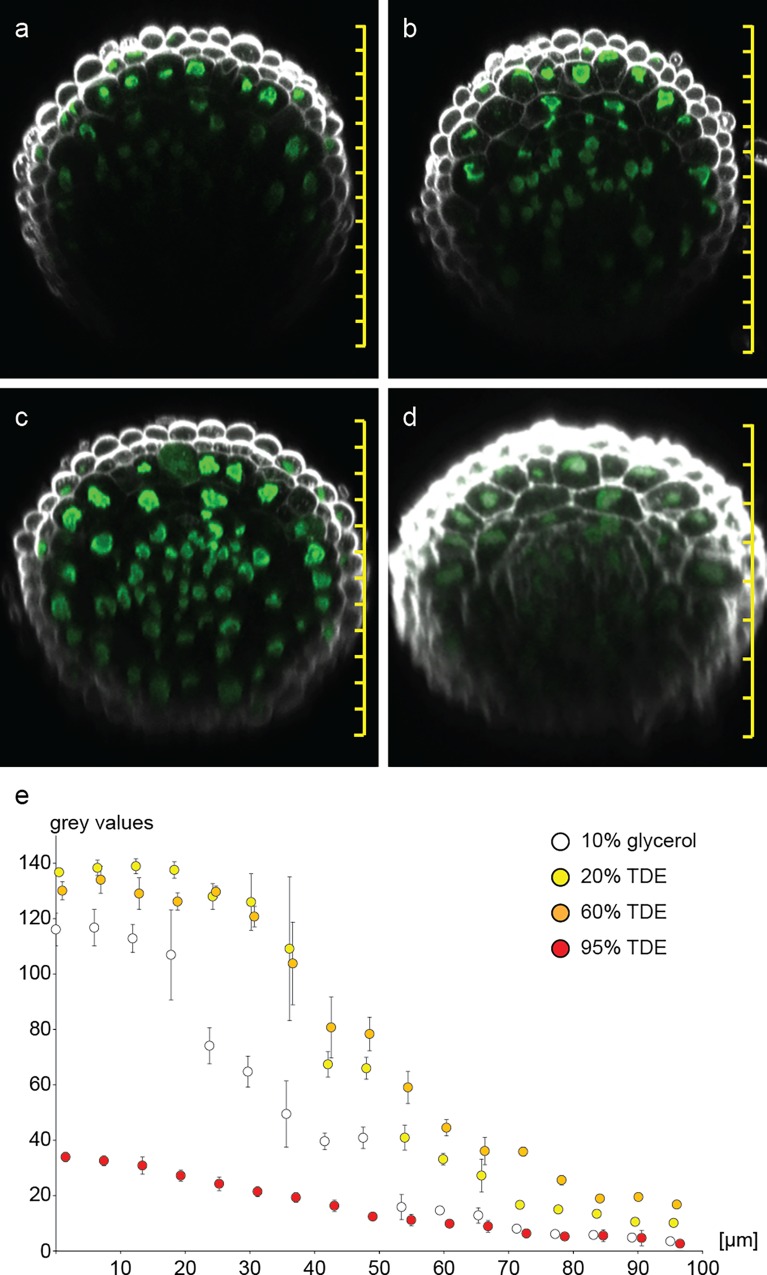
Increasing fluorescence imaging depth in roots. Orthogonal sections through roots expressing *pCWC15*:*CWC15-3xeGFP* (green) were reconstructed from z-stacks for (a) 10% glycerol, (b) 20% TDE, (c) 60% TDE and (d) 95% TDE. Cell walls were stained with SR2200 (white). The decline of fluorescence intensity from top to bottom across the root is plotted as 8 bit grey values of descending nuclei for the corresponding clearing solutions (e). Error bars indicate standard deviation of nuclear grey values from n = 3 roots. Scale bars = 20μm.

We performed a similar experiment with root tissue ubiquitously expressing nuclear-localized GFP (pCWC15::CWC15-eGFP). For root tissue, fixation times of 1h were already sufficient to preserve tissue integrity and fluorescent protein activity and allowed the use of up to 70% TDE for clearing (Fig 4). Similar to our observation in cotyledons, 95% TDE lead to tissue softening and shrinkage as well as reduction of FP activity in roots (Fig 4D). As expected, the signal intensity of eGFP decreased with increasing focal depth in all samples to various degrees. Generally speaking, TDE embedding resulted in tissue clearing and allowed deeper penetration of the tissue, a result consistent with recent work [24]. This effect depends on the TDE concentration, so that deeper imaging is possible in 60% TDE than in 20% TDE. In 95% TDE the signal shows the lowest reduction with increasing focal depth, but it is already dim at the beginning.

When comparing roots with cotyledons, we would conclude that different plant structures respond differently to TDE and that roots can tolerate higher TDE concentrations after shorter tissue fixation. To further test the effect of TDE on a more delicate tissue, we observed nuclear-localized eGFP in young Arabidopsis ovules after different incubation times in 70% and 95% TDE (S3 Fig). We deliberately fixated the tissue for only 5 min. Consistent with the results obtained in leaf and root tissue, we observed tissue shrinkage deforming the ovules, especially at high TDE concentrations (S3D and S3G Fig). This was especially pronounced due to the short fixation time (compare S3 Fig and Fig 1).

**Fig 5:**
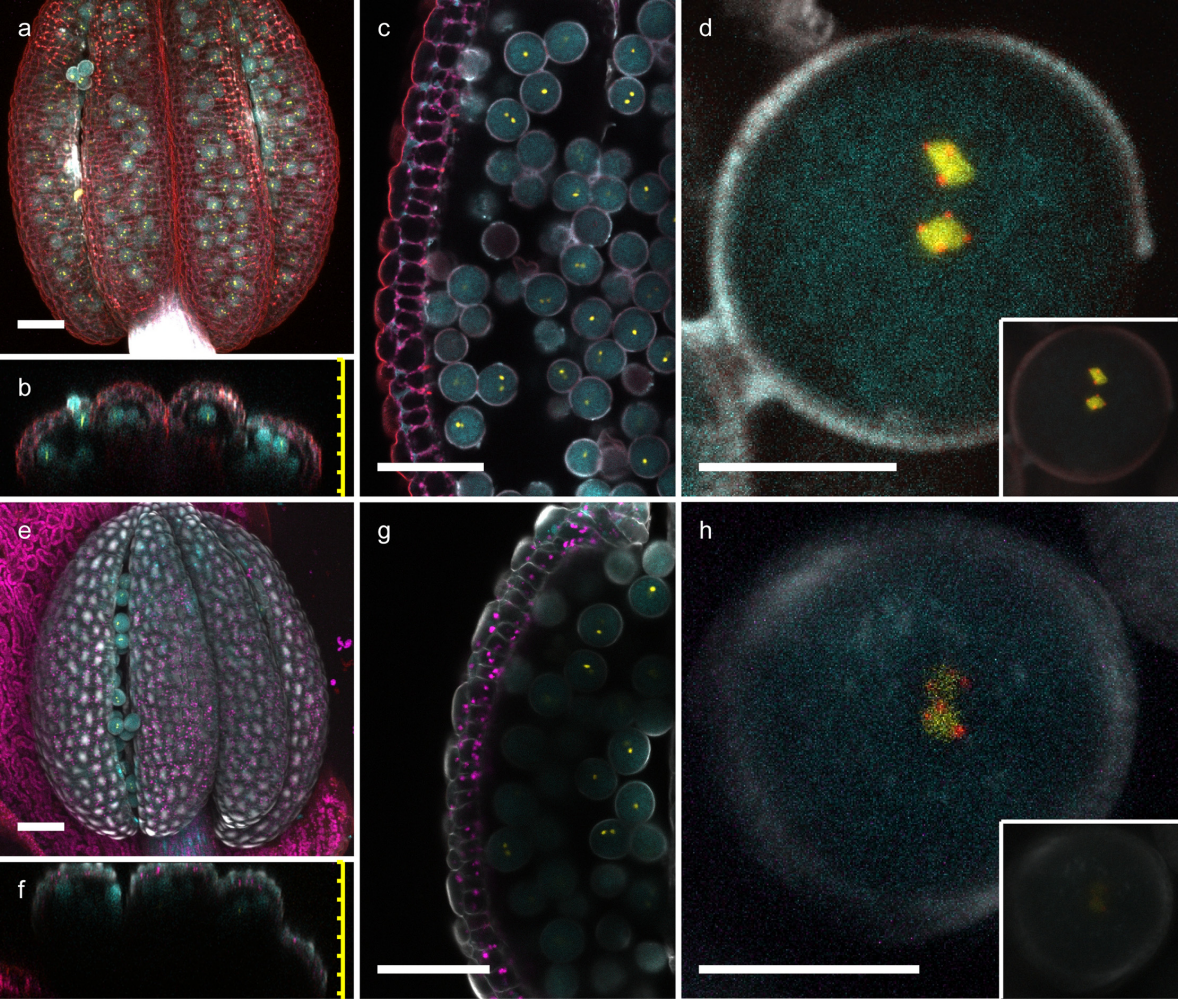
TDE clearing of anthers and pollen. Anthers of transgenic plants expressing three different, pollen-specific fluorescent markers. pLAT52::er-2xCFP labels the endoplasmic reticulum of the vegetative cell, pHTR10::HTR10-2xYFP marks chromatin of the sperm cells, while pHTR12::HTR12-mCherry highlights centromeres in the sperm cell nuclei. Maximum projections (a, e) and orthogonal sections (b, f) are shown for TDE (a-d) and 10% glycerol (e-h). Pollen was imaged through the surrounding anther tissue (overview in c and g; magnification in d and h). The fluorescence signal in pollen is much brighter in TDE-embedded tisuue (d) in comparison to 10% glycerol (h) when using the same imaging settings (inlay). CFP signal shown in cyan, YFP in yellow, mCherry in red; background staining with SR2200 is shown in grey scale. Scale bars = 50 μm (a-c, e-g) and 10 μm (d, h), respectively.

As in the previous experiments, 95% TDE caused strong tissue shrinkage and loss of FP activity (S3G–S3J Fig). This experiment further emphasizes the importance of proper tissue fixation when embedding in high TDE concentrations. As an additional delicate tissue, we used anthers of transgenic plants expressing three different fluorescent proteins in mature pollen (ER-localized CFP in the veg. cell as well as HTR10-YFP and HTR12-mCherry in sperm cells; Musielak et al. 2015). Based on our previous results, anther tissue was fixated overnight and incubated overnight in 70% TDE and 10% glycerol, respectively. Under these fixation and incubation conditions, no tissue deformation was noticeable and all three fluorophores seemed unaffected by TDE (Fig 5). Maximum projections and orthogonal sections show that TDE embedding allows to image pollen grains inside the anther, while the anther tissue can only be penetrated at anther apertures when embedded in 10% glycerol (Fig 5A, 5B, 5E and 5F and S1 Movie). TDE embedding lead to more defined images of pollen, allowing the clear detection of centromere-localized mCherry while glycerol embedding resulted in relatively blurred images (Fig 5D and 5H). The increased tissue clearing by TDE embedding also results in a stronger fluorescent signal in comparison to glycerol embedding when using identical imaging settings (Fig 5D and 5H inlay).

Taking these results together, special attention should be paid to fixation of the tissue before clearing with TDE as insufficient fixation will lead to tissue disintegration and shrinkage as well as partial or complete loss of fluorescent protein activity. Our data suggest that overnight fixation and TDE concentrations of 50–70% would be a good compromise for most tissue types. Stronger fixation might allow even for tissue incubation in higher TDE concentrations, theoretically giving even better clearing results. On the other hand, if rapid sample processing is desired, shorter fixation times can be compensated by reducing the TDE concentration to around 20–30%, but this will lead to reduced tissue clearing.

### TDE makes challenging tissues accessible for two-photon microscopy

**Fig 6:**
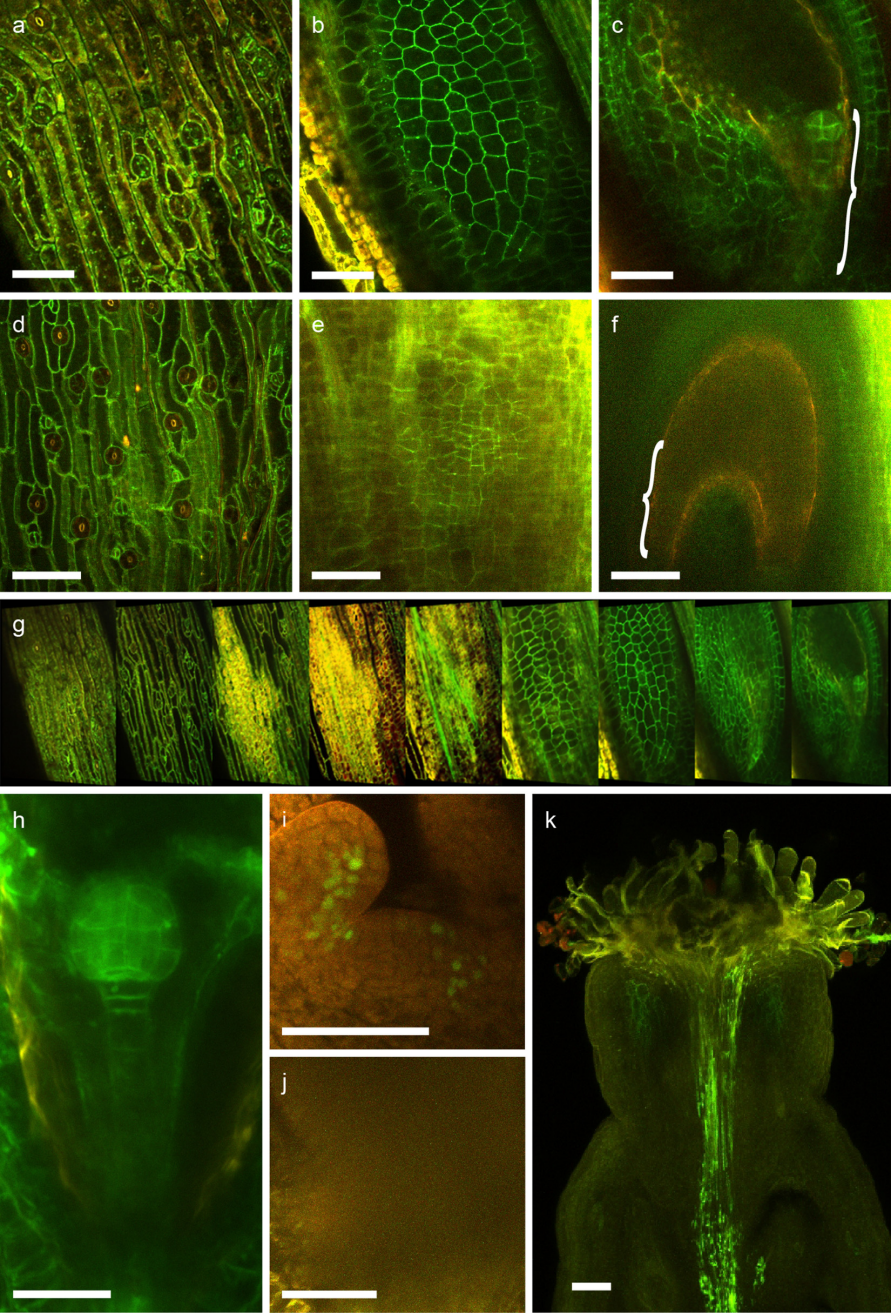
Two-photon microscopy of Arabidopsis siliques and shoot apexes. a-g) Optical sections through a silique expressing plasma membrane-residing eGFP-SYP132, showing silique epidermis (a, d), seed epidermis (b, e) and section through ovules containing 16-cell embryos (c, f; bracket indicates position of the embryo) after TDE (a-c) or glycerol embedding (d-f). An overview of scans through a silique cleared by TDE embedding containing ovules with 16-cell stage embryo is shown in g). Imaging of embryos through the silique can be achieved after TDE treatment even for late globular stage embryos (h). Two-photon images of shoot apexes of 5 day old seedlings expressing pWOX2::WOX2-eGFP after clearing in 70% TDE (i) or 10% glycerol (j). Two-photon image of a silique with pollen tubes expressing membrane-localized PEN1-YFP (k). eGFP and YFP signals are shown in green, autofluorescence is shown in red. Scale bars = 50 μm.

Two-photon microscopy has become the method of choice for deep tissue imaging as it minimizes the problem of light scattering. TDE has significantly reduced absorption of near-infrared light in comparison to water, a fact that should provide an additional advantage for two-photon microscopy [22]. To test this, we tried to image developing embryos in situ inside ovules residing inside the fruit tissue by scanning through a whole silique that was previously fixated and incubated for three hours in 70% TDE or 10%glycerol, respectively (Fig 6). This tissue is especially challenging as there are several maternal cell layers that cause disturbance of light transmission, making the tissue basically non-transparent. For realistic evaluation of clearing, we chose eGFP-tagged SYP132 expressed under its own promoter which uniformly labels the plasma-membrane with weak to moderate fluorescence intensity [28]. Fig 6 shows the epidermis of the silique (a), the epidermis of an ovule (b) and a 16-cell embryo inside the ovule (c). This demonstrates that deep penetration of tissue is possible by TDE embedding as we were able to detect fluorescence marker-labelled embryos within a whole silique through all surrounding layers (Fig 6C and 6G and S2 Movie), which was impossible for us with this relatively weak marker line when using glycerol as embedding medium (Fig 6D–6F). We also imaged nuclear-localized WOX2-GFP in the shoot apical meristem of 5 day-old seedlings without prior removal of surrounding leaf primordia (Fig 6I and 6J). As mentioned above, the tissue was fixated and incubated for 3h in 70% TDE or 10% glycerol, respectively. While it was possible to penetrate surrounding primordial tissue and get clear images of the shoot apical meristem with TDE-embedded samples (Fig 6I), similar microscope settings lead to blurry undefined images in 10% glycerol (Fig 6J). Taken together, our data indicate that TDE embedding is superior to 10% glycerol also for 2-photon microscopy.

To test this application further, we pollinated a pistil with pollen expressing membrane-localized pLAT52:YFP-PEN1 and used TDE embedding to visualize growing pollen tubes. 24 hours after pollination, the samples were fixated and incubated in 70% TDE for three hours each. TDE clearing made it possible to trace pollen tubes within the pistil, which would be difficult or not possible with standard clearing methods (Fig 6K and S3 Movie). These examples illustrate that TDE is a powerful clearing agent to study developmental processes that are hidden by several surrounding tissue layers as this is the case for example during plant reproduction.

Taken together, our results demonstrate that TDE in concentrations around 50–70% is a versatile embedding medium for laser scanning confocal microscopy and two-photon microscopy, which in many applications might be superior to the widely used 10% glycerol solution or water.

## Discussion

Our in vitro results suggest that commonly used FPs, such as GFP, YFP and RFP variants, can be employed in TDE concentrations close to 100%, albeit with strongly reduced fluorescence intensity in concentrations above 95%. In this experiment, the fluorescent activity seemed to increase in concentrations around 80%, which is consistent with previous reports that showed that the behaviour of different FPs can be astonishingly variable in TDE [25]. The tested FPs in this experiment were still active even in 99% TDE, so that a concentration of 95%, perfectly matching the RI of commonly-used immersion oil, could theoretically be used. It was suggested that red fluorescent proteins are less sensitive to high TDE concentrations [25]. In our experimental setup, there was no positive effect for mCherry over Citrine-YFP at 95% or 99% TDE. Both FPs however, seem to be more stable in these high TDE concentrations than eGFP or Venus-YFP. Especially eGFP showed strongly reduced fluorescence in TDE concentrations higher than 70%. We also tested for auto-fluorescence of TDE in vitro and could not measure any significant levels of autofluorescence.

In contrast to the in vitro results, we experienced that in situ high concentrations of TDE (95%) seem to quench the fluorescence of FPs in cotyledons, seed and root samples. In an elegant experiment, Aoygi et al. showed that the fluorescence of TDE can be restored, when samples are washed and immersed in a lower concentration of TDE afterwards. The authors draw the conclusion that a depletion of the fluorescent proteins cannot be the reason and speculated that the fluorophore itself is affected by TDE [22]. In our experiment however, it appears that the plant tissue itself is softened and loses structural integrity in high TDE concentrations and after long-term incubation. The diffusion of chlorophyll out of chloroplasts as well as leakage of nuclear-localized tdtomato out of nuclei would indicate that membrane structures are disintegrated by TDE. This negative effect was not reported in previous plant studies [24] but is well described for clearing of animal tissue [29] and emphasizes the need of sufficient tissue fixation before clearing. Together with the results from the measurement of solved proteins in TDE and reports in literature that clear detection of fluorescence in 95% TDE is possible [30], we hypothesize that the lower fluorescence intensity in 95% TDE is not necessarily an effect of direct quenching of the FP. More likely, it is a consequence of the loss of membrane integrity causing pH changes or the release of vacuolar proteases. As general protocol for most tissue types, we recommend overnight fixation at 4°C before overnight clearing in 50–70% TDE, depending on the fluorescent protein and tissue type of choice (Table 1). This preserves tissue integrity and FP activity while still maintaining the advantages of TDE with a RI up to 1.47 [25]. Delicate tissue might be imaged already after 3h incubation in TDE. If a more rapid protocol is desired, fixation time and TDE concentration as well as incubation time can be reduced (Table 1). However, this leads to less pronounced tissue clearing.

**Table 1:** Recommended tissue fixation and TDE concentrations for clearing of various tissue types.

Tissue-type	Standard clearing protocol		Rapid clearing protocol	
	o/n fixation, 3h - o/n clearing		1h fixation, 1h clearing	
	CFP / GFP	YFP / dsRed	CFP / GFP	YFP / dsRed
Root				
Shoot	70% TDE	70% TDE	40% TDE	50% TDE
Cotyledons				
Leaves	50% TDE	50% TDE	20% TDE	20% TDE
Anthers				
Siliques	50% TDE	70% TDE	40% TDE	50% TDE
Isol. ovules				

Depending on the tissue type of choice and fixation time, the table gives recommendations for TDE concentrations (in % v/v in H2O) for optimal clearing while at the same time preserving tissue integrity.

We tested the application of TDE for different plant tissues and different microscope techniques. TDE proved to have an advantage over glycerol in single-photon microscopy by allowing for deeper tissue penetration in tissues covering various parts of the plant, such as roots, leaves, anthers with pollen, and isolated ovules. At a depth of e.g. 40 μm, FPs in 60% TDE show roughly two times higher fluorescence values than FPs in 10% glycerol in root tissue (Fig 4E). While TDE embedding proves to be useful for single-photon microscopy and allows for scanning through thick and opaque tissues, it seems especially valuable for two-photon microscopy. Since there is no pinhole excluding out-of-focus light in 2-photon microscopy, scattering of the excitation laser immediately causes blurring of the image and also reduces the efficiency of 2-photon excitation. Reducing light scattering in the infrared spectrum therefore leads to brighter and more focused images. Furthermore, TDE has a lower absorption of near-infrared light than water, theoretically giving a further advantage for 2-photon microscopy over aqueous embedding media.

As an example of using TDE in 2-photon microscopy, we took images of pistils with pollen tubes marked by a reporter construct. Clearing with TDE makes it possible to follow pollen tubes within the pistil, a feature which could be used to visualize the fertilization process. The relatively fast and simple preparation could even enable researchers to perform high-throughput experiments of pollen tube growth within the pistil such as genetic screens. A similar experiment was done with ClearSee [19]. The treatment with this clearing agent resulted in outstanding images of multi-coloured pollen tubes growing inside the pistil. For the high-resolution images however, samples were treated with ClearSee for at least 2 weeks. While our TDE-based clearing protocol did not result in such stunning images as shown by Kurihara et al (2015), clearing of the tissue can be achieved within a few hours. This illustrates that TDE-treated samples can be used for microscopy very rapidly and therefore allow a fast experimental setup that lends itself also for high-throughput experiments. For long-term treatments with the demand of highly accurate images on the other hand, ClearSee would probably be the clearing method of choice [19].

Besides its direct advantageous features in general microscopy, we could also show that TDE provides a small advantage in the reduction of photo-oxidation. However, this effect seems to be specific on the particular FP, a result that is consistent with existent reports, which show that FPs seem to be affected in a different manner by TDE [22, 25, 30]. Furthermore, this effect is rather weak and it is therefore questionable if it makes a noticeable difference in most imaging experiments.

In summary, tissue fixation is a necessary prerequisite for TDE clearing. If this is taken into account, TDE embedding can offer significant advantages in clearing and optimization of the sample’s RI. Furthermore, TDE is easily applicable, flexible, non-toxic and inexpensive.

## Materials and Methods

### Plant material and transgenic lines

Arabidopsis plants (Col-0 ecotype) were grown under long-day conditions as described before [31]. The transgenic *pLAT52*:*YFP-PEN1* line was kindly supplied by Sandra Richter and Gerd Jürgens. The *pSYP132*:*GFP-SYP132* and the *pS4*:*n3xGFP* lines have been described previously [28, 32]. The multi-color pollen marker has been obtained by crossing individual transgenic lines and has been described before (Musielak et al., 2015).

### Plasmid construction

A *RPS5a* promoter fragment was PCR amplified using primers TTTTTGGCGCGCCATAATCGTGAGTAGATAT and TTTTTATTTAAATTGGGTACCCGGCTGTGGT and digested with AscI and SwaI to replace a *NTA* promoter fragment with similar restriction sites in *GIIb pNTA>>NLS-tdtomato* [33]. For *pCWC15*:*CWC15-3xGFP*, a 2 kb promoter fragment of *CWC15* was PCR amplified and ligated into pGreen containing *NLS-3xGFP*. The coding region of *CWC15* was PCR amplified and ligated in-frame into the previous vector.

### Cloning of expression vectors and purification of fluorescent proteins

The coding regions of fluorescent proteins were amplified by PCR with attached restriction sites for BamHI and EcoRI. The amplified sequences were ligated into a likewise digested expression vector containing a 6-fold His-Tag (gift from Ole Herud). E. coli DH5α cells were grown on LB plates, transferred to 2ml binding buffer and sonicated with a Bandelin Sonicator. FPs were purified with His SpinTrap columns and NAP(TM)-25 Sephadex G-25 DNA grade columns (both GE Healthcare) according to the manufacturer’s protocol and solved in MOPS buffer (Roth).

### Measurement of in vitro fluorescence

Protein concentration in MOPS buffer was measured with a Spectrophotometer at 280 nm (Nano Drop 1000 Photospectrometer) and for all fluorophores adjusted to a similar level and fluorescence was measured with a Tecan plate reader after 10 min incubation in the respective TDE concentrations. mCherry was excited at 560nm and emission was measured at 610 nm. eGFP, Venus-YFP and Citrin-YFP were excited at 495nm with a bandwidth of 10nm and measured at 535nm with a bandwidth of 25nm. The fluorophores in water, TDE and chloral hydrate, respectively, were measured in triplicates.

### TDE clearing

If not indicated otherwise, samples were first fixated in 4% paraformaldehyde (PFA) at room temperature for 1h after brief vacuum infiltration (80 mbar; as described previously; Musielak et al., 2015), washed with water and then directly transferred to 10% glycerol or the desired TDE concentration and incubated at room temperature for 1h before microscopic imaging.

### Brightfield transmission and in vivo fluorescence measurements

Brightfield images of seeds embedded in the various clearing solutions were taken on a Zeiss AxioImager Z.1 microscope equipped with a Plan-Apochromat 20x/0,8 using a Zeiss Axiocam HRc Rev.3 and Zeiss AxioVision imaging software. Average grey values of entire seeds and background were measured using ImageJ 1.49 and transmission in percent was calculated. *pCWC15*:*CWC15-3xGFP* transgenic roots were fixated and stained as described previously [34]. eGFP fluorescence in *Arabidopsis* roots for imaging depth measurements was recorded on a Leica TCS SP8 confocal microscope equipped with a HC PL APO 40x/1.10 W Corr CS2 lens. Images were taken in xy planes with z-stacks through the entire root. Orthogonal sections were reconstructed in ImageJ and 8 bit grey values for every pixel of successive nuclei from the second or third outermost cell layer were measured from top to bottom of the root image.

For the evaluation of fixation time, incubation time and TDE concentrations in leaf tissue, *pRPS5a>>NLS-tomato* cotyledons were fixated in 4% PFA for a specific time as described previously (Musielak et al., 2015) and incubated in various TDE concentrations or 10% glycerol for 1h and overnight. Tdtomato fluorescence and background fluorescence in the far-red spectrum were recorded on a Leica TCS SP8 confocal microscope equipped with a HC PL APO 40x/1.10 W Corr CS2 lens. Images were taken in xy planes with z-stacks through the entire leaf (around 180 µm depth). Orthogonal sections were reconstructed in Fiji.

### Bleaching experiments

Bleaching experiments were performed in Arabidopsis roots on an Olympus FV1200 confocal microscope equipped with a UPlanSApo 40x objective using a 480nm Argon laser (60% laser power, PMT 500V) and a 559nm Diode laser (20% laser power, PMT 380V) for nuclear GFP and ntdtomato, respectively. Using continuous excitation in a xyt free run scan, images of epidermal cells were taken every 3.26 s. Regions of interest (ROIs) corresponding to fluorescent nuclei were selected using ImageJ 1.49v and relative, average grey values of nuclei at the different time points for different TDE concentrations as well as 10% glycerol were measured and calculated.

### Two-photon microscopy

Two days after pollination, siliques expressing *pSYP132*:*GFP-SYP132* and 24 h after pollination with *pLAT52*:*YFP-PEN1*, wild-type siliques were collected, respectively, and after fixation cleared at room temperature for 3h. Similarly, shoot apexes of pWOX2::WOX2-GFP seedlings were collected 5 days after germination and fixated for 3 h followed by 70% TDE incubation for 3 h. Images were taken with a Zeiss LSM 780 NLO equipped with a two-channel non-descanned detector for two-photon imaging using a MaiTai DeepSee eH laser and LD LCI Plan-Apochromat 25x/0,8 Imm Korr DIC or LD C-Apochromat 40x/1,1 W Korr objectives, respectively.

## Supporting Information

S1 Fig Stability of fluorescence activity of FPs incubated in TDE over time. Fluorescence of heterologously expressed and purified FPs was measured in vitro for one hour. eGFP (blue line), Venus YFP (green line), Citrine YFP (yellow line) and mCherry (red line) were incubated in water (a), 20% TDE (b), 50% TDE (c), 70% TDE (d), 95% TDE (e), and 99% TDE (f).
(TIF)

S2 Fig Bleaching of FPs in root epidermal cells. Starting at the same absolute fluorescence intensity, relative fluorescence in percent corresponding to grey values of root epidermal nuclei was plotted over time (number of CLSM scans) and different clearing solution concentrations.
(TIF)

S3 Fig Effect of TDE on overall structure and fluorescence in early ovules after 1h and 24h incubation. DIC images (left column) and images of zygotes or embryos expressing *pS4*:*n3xGFP* (middle and right column) incubated 1h (left and middle column) or 24h (right column) in different clearing solutions. (A-C) 10% glycerol, (D-F) 70% TDE and (G-I) 95% TDE. Fixation time 5 min. Curved brackets indicate size and position of zygote or embryo. Scale bars = 50 µm.
(TIF)

S1 Movie 3D reconstruction of TDE-cleared anthers. (AVI)

S2 Movie Optical sections through a silique expressing *pSYP132*::*GFP-SYP132*. (AVI)

S3 Movie 3D reconstruction of silique pollinated with *pLAT52*::*PEN1-YFP* pollen. (AVI)
