# Supplementary figures and images for: A Versatile Optical Clearing Protocol for Deep Tissue Imaging of Fluorescent Proteins in Arabidopsis thaliana
Source: PLoS One. 2016 Aug 12;11(8):e0161107. doi: 10.1371/journal.pone.0161107 (PMC4982668; doi:10.1371/journal.pone.0161107)

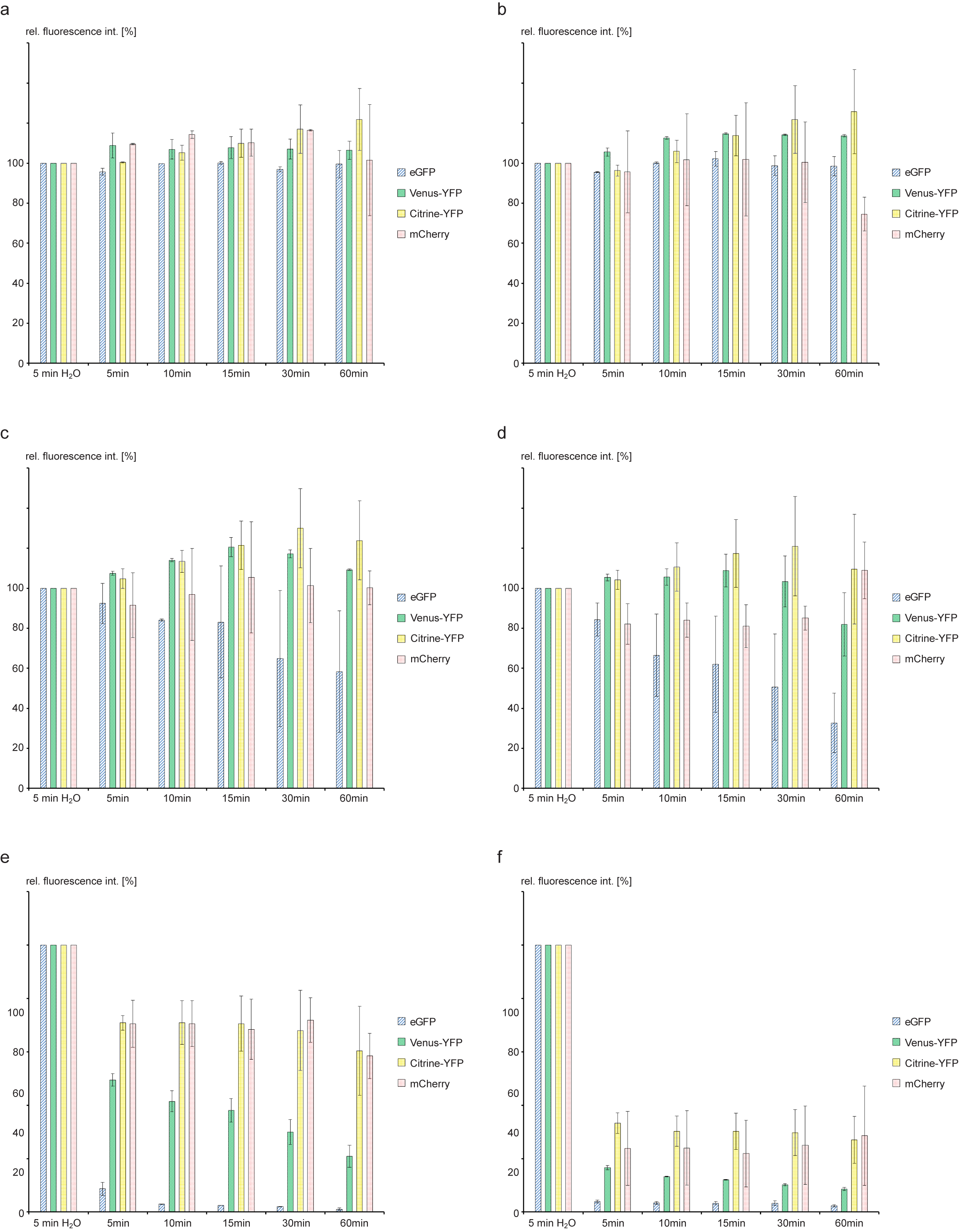

Supplement: S1 Fig — Fluorescence of heterologously expressed and purified FPs was measured in vitro for one hour. eGFP (blue line), Venus YFP (green line), Citrine YFP (yellow line) and mCherry (red line) were incubated in water (a), 20% TDE (b), 50% TDE (c), 70% TDE (d), 95% TDE (e), and 99% TDE (f). (TIF) [file pone.0161107.s001.tif]

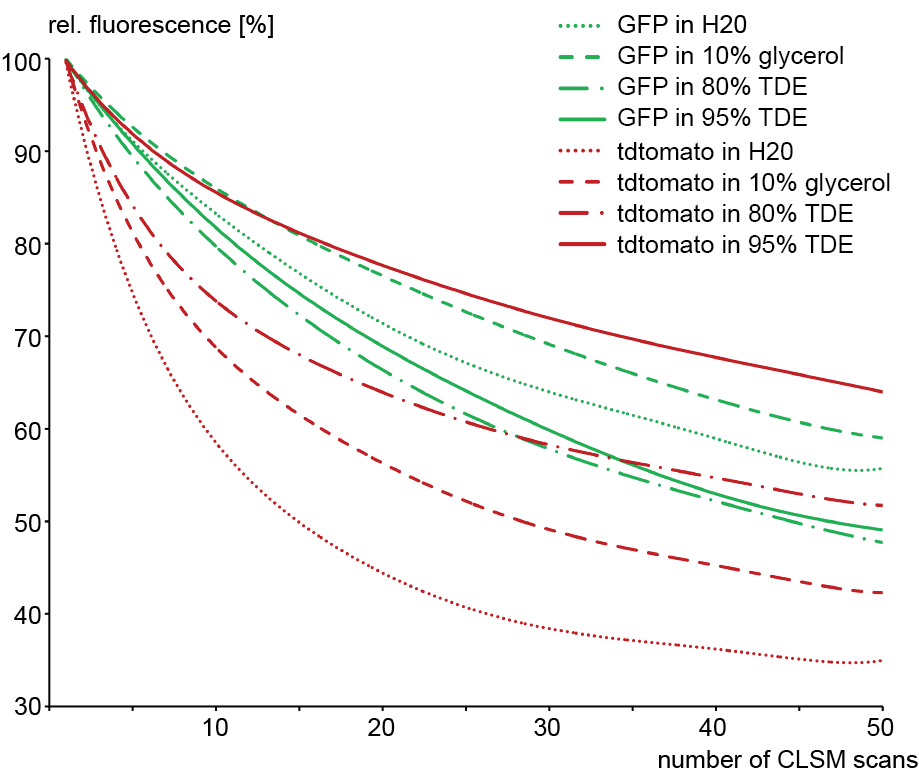

Supplement: S2 Fig — Starting at the same absolute fluorescence intensity, relative fluorescence in percent corresponding to grey values of root epidermal nuclei was plotted over time (number of CLSM scans) and different clearing solution concentrations. (TIF) [file pone.0161107.s002.tif]

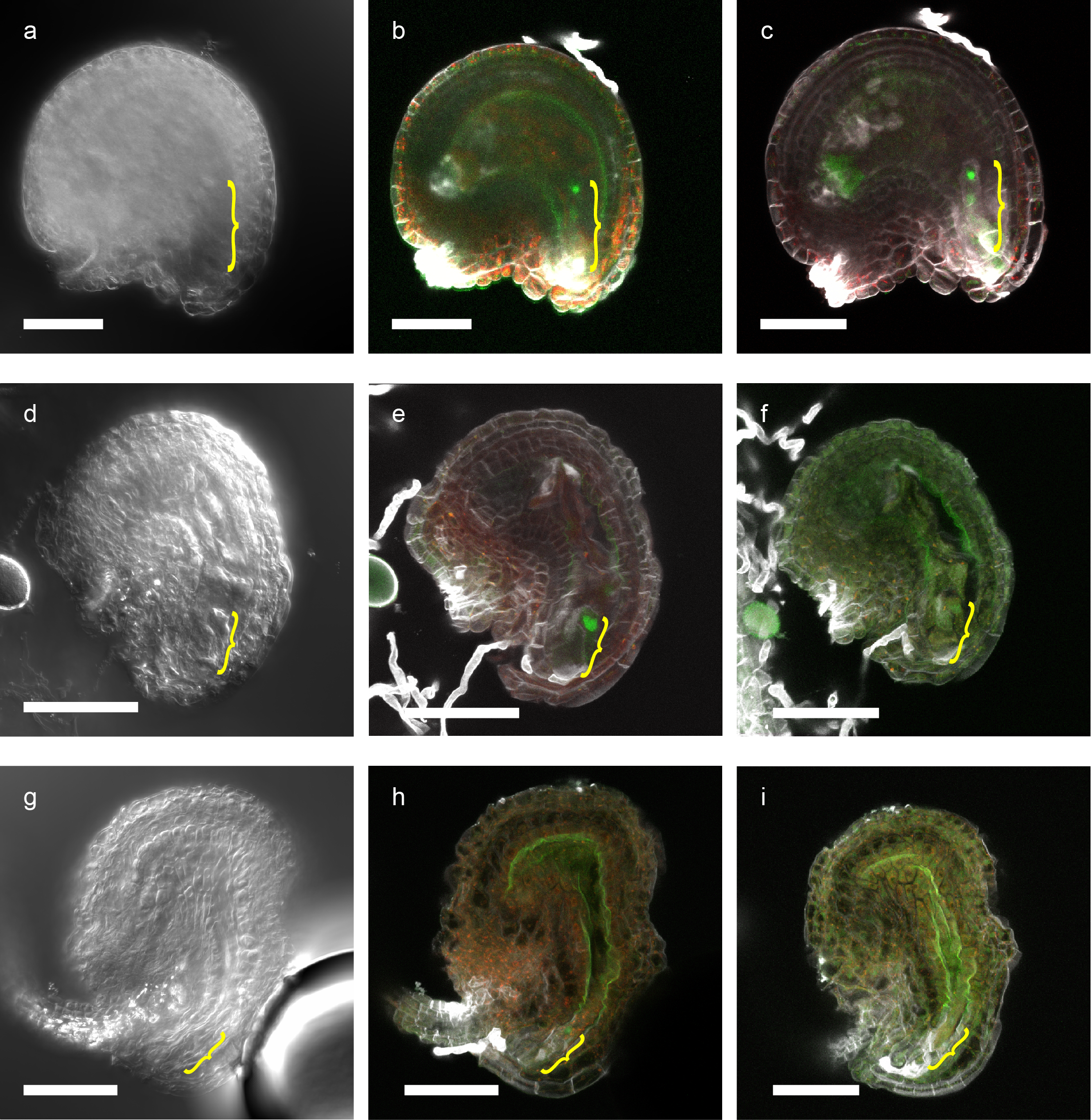

Supplement: S3 Fig — DIC images (left column) and images of zygotes or embryos expressing pS4:n3xGFP (middle and right column) incubated 1h (left and middle column) or 24h (right column) in different clearing solutions. (A-C) 10% glycerol, (D-F) 70% TDE and (G-I) 95% TDE. Fixation time 5 min. Curved brackets indicate size and position of zygote or embryo. Scale bars = 50 µm. (TIF) [file pone.0161107.s003.tif]
